# Fish-Derived Protein Hydrolysates Increase Insulin Sensitivity and Alter Intestinal Microbiome in High-Fat-Induced Obese Mice

**DOI:** 10.3390/md21060343

**Published:** 2023-06-02

**Authors:** Maria G. Daskalaki, Konstantinos Axarlis, Antiopi Tsoureki, Sofia Michailidou, Christina Efraimoglou, Ioanna Lapi, Ourania Kolliniati, Eirini Dermitzaki, Maria Venihaki, Katerina Kousoulaki, Anagnostis Argiriou, Christos Tsatsanis

**Affiliations:** 1 Laboratory of Clinical Chemistry, Medical School, University of Crete, 70013 Heraklion, Greece; m.daskalaki@med.uoc.gr (M.G.D.); konax@outlook.com (K.A.); christinaefr96@gmail.com (C.E.); iwanna_lapi@hotmail.com (I.L.); raliakolliniatis21@gmail.com (O.K.); renaderm@med.uoc.gr (E.D.); venihaki@med.uoc.gr (M.V.); 2 Institute of Molecular Biology and Biotechnology, FORTH, 71100 Heraklion, Greece; 3 Institute of Applied Biosciences (INAB), CERTH, Thermi, 57001 Thessaloniki, Greece; adatsoureki@certh.gr (A.T.); sofia_micha28@certh.gr (S.M.); argiriou@certh.gr (A.A.); 4 Department of Nutrition and Feed Technology, Nofima AS, 5141 Bergen, Norway; katerina.kousoulaki@nofima.no; 5 Department of Food Science and Nutrition, University of the Aegean, Myrina, 81400 Lemnos, Greece

**Keywords:** obesity, fish, microbiome, diabetes, protein hydrolysates

## Abstract

Obesity and type 2 diabetes are characterized by low-grade systemic inflammation and glucose intolerance, which can be partially controlled with nutritional interventions. Protein-containing nutritional supplements possess health-promoting benefits. Herein, we examined the effect of dietary supplementation with protein hydrolysates derived from fish sidestreams on obesity and diabetes, utilizing a mouse model of High-Fat Diet-induced obesity and type 2 diabetes. We examined the effect of protein hydrolysates from salmon and mackerel backbone (HSB and HMB, respectively), salmon and mackerel heads (HSH and HMH, respectively), and fish collagen. The results showed that none of the dietary supplements affected weight gain, but HSH partially suppressed glucose intolerance, while HMB and HMH suppressed leptin increase in the adipose tissue. We further analyzed the gut microbiome, which contributes to the metabolic disease implicated in the development of type 2 diabetes, and found that supplementation with selected protein hydrolysates resulted in distinct changes in gut microbiome composition. The most prominent changes occurred when the diet was supplemented with fish collagen since it increased the abundance of beneficial bacteria and restricted the presence of harmful ones. Overall, the results suggest that protein hydrolysates derived from fish sidestreams can be utilized as dietary supplements with significant health benefits in the context of type 2 diabetes and diet-induced changes in the gut microbiome.

## 1. Introduction

Modern lifestyle changes have contributed to the increasing prevalence of a variety of chronic diseases, including metabolic syndrome and type 2 diabetes mellitus. At the basis of the latter typically stand obesity and insulin resistance, the incidence of which has been growing into a pandemic since the early 1980s [1]. A number of factors may be implicated with varying significance, including genetics [2]. However, lifestyle choices, such as dietary habits and physical (in)activity, heavily determine the likelihood of developing the above conditions [3].

Interestingly, the gut microbiome has recently been shown to actively modulate the host’s metabolism, probably playing a crucial role in the emergence and pathogenesis of obesity and insulin resistance [4]. It may also modify the levels of inflammation, which contributes to metabolic dysregulation, through a number of mechanisms, including metabolite-mediated host immune system modulation and maintenance of the gut’s barrier integrity [5].

Dietary interventions are at the forefront of managing and combating obesity, diabetes, and metabolic syndrome. Nutritional supplements play a useful and supportive part in dietary interventions for reasons that include their content of bioactive compounds, nutrients, minerals, and essential trace elements [6]. Fish are rich in proteins and bioactive peptides, minerals, vitamins, as well as omega-3 fatty acids and phospholipids, just to name a few, rendering them an appealing source of high-quality nutrition. Indeed, dietary supplements of fish origin have been suggested to exert a positive effect on conditions such as obesity and diabetes. Fish-derived oil, being a great source of ω-3 polyunsaturated fatty acids (ω-3-PUFAs), has been exhibited to ameliorate the obese phenotype [7], whereas a variety of fish extracts developed from tuna [8], Tapra fish [9] or Masou salmon [10], also indicate an anti-obesity action. However, the knowledge regarding whether and in which way the gut microbiome is shaped by fish extracts is very sparse. Furthermore, the potential contribution of fish-derived collagen in suppressing weight gain [11] and ameliorating obesity has been noted in mice, partially through inhibiting differentiation of preadipocytes to mature adipocytes [12]. Notably, fish collagen is an attractive alternative to mammalian, as the latter might present some drawbacks, such as concerns related to disease transmission and allergic reactions, while vegan collagen does not have the original structure of the naturally produced complex protein [13]. However, no data exist on the possible synergistic action of fish-derived collagen with fish protein hydrolysates on obesity and diabetes.

Each year, large amounts of sidestreams are generated by the fish industry. It is estimated that more than 50% of fish biomass is widely considered “waste”, including fins, heads, skins, and viscera, or used for low-value applications, such as animal nutrition and fertilizers. In addition, by-catch products, fish species, and undersized or damaged fish with low or no commercial value are considered fish waste, not only reducing economic growth but also posing a threat to aquatic ecosystems [14]. Efforts to reduce bio-waste led to the valorization of fish sidestreams in order to produce high-value commercial compounds such as enzymes, peptides, collagen, chitin, polyunsaturated fatty acids (PUFAs), and bioactive compounds [14,15]. Among valorization methods, the generation of bioactive peptides through fish sidestream protein hydrolysis has gained great interest in recent years, releasing bioactive peptides with anti-microbial, antiproliferative, and antioxidant properties [16].

The aim of the present study was to investigate the potential effects of fish-derived protein hydrolysates used as dietary supplements, including fish collagen, on obesity and insulin resistance, using the murine model of diet-induced obesity and type 2 diabetes, while also considering the impact of these dietary supplements on the composition of the gut microbiome.

## 2. Results and Discussion

### 2.1. Nutritional Supplement Preparation

Fish-derived hydrolysates (Table 1) were generated using mackerel and salmon sidestreams aiming to investigate their possible bioactivity in high-fat-induced obesity in mice and their potential use as nutritional supplements in obese-related pathological conditions. The composition of the different supplements and their organoleptic properties have been recently described [17].

### 2.2. Fish-Derived Nutritional Supplement HSH Induces Insulin Sensitivity in High-Fat-Induced Obese Mice

**Figure 1:**
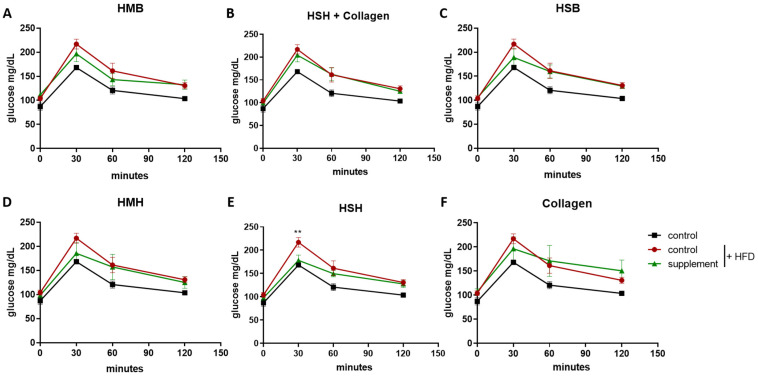
Monitoring the effect of fish-derived hydrolysates in high-fat diet-induced insulin resistance. (**A**–**F**) Mice were fed with the indicated nutritional supplements and then subjected to a glucose tolerance test. Black color indicates the group of mice consuming the lean diet, and red color indicates the group of mice consuming the high-fat diet used as control. Graphs represent mean ± SEM. A 2-way ANOVA test was performed. ** *p* < 0.01.

It has been previously reported that fish-derived supplements exhibit beneficial properties, helping in the management of chronic diseases such as cardiovascular disease, type 2 diabetes, and autoimmunity by ameliorating insulin resistance, obesity, inflammation, and muscle damage as well as modulating gut microbiota [18,19]. The majority of basic and clinical research has been focused on the extraction and administration of fish-derived oils containing high amounts of omega-3 fatty acids, eicosapentaenoic acid (EPA), and docosahexaenoic acid (DHA) [20,21]. In this study, we aim to investigate the potential beneficial role of nutritional supplements containing fish sidestream-derived protein hydrolysates, which are rich in bioactive peptides. To test the bioactivity of the aforementioned fish-derived supplements in High-Fat Diet-induced (HFD) insulin resistance, we treated mice for 3.5 weeks with HFD supplemented with 5% *w*/*w* of each protein hydrolysate. In each diet group, 5 mice of both sexes were randomly allocated. As a control, a 5% soy protein supplement was used to simulate protein intake levels, as fish supplements contain large amounts of protein. Additionally, a group of mice consuming a control diet (lean) supplemented with 5% of soy protein was used to monitor the progress of the experiment. Animal weight was monitored weekly, and a glucose tolerance test was performed at the end of the experiment (Figure 1). Interestingly, the group consuming the HSH nutritional supplement exhibited decreased glucose intolerance (Figure 1E); in fact, 30 min post the dextrose injection, glucose levels in blood highly resembled those consuming a lean diet. Moreover, it is worth noting that both sexes were present in this study, as it is known that sex-related differences concerning insulin sensitivity may occur [22]. Notably, when only male mice were considered, the HMB diet-consuming group exhibited significantly reduced insulin resistance compared to high-fat diet mice (Supplementary Figure S1). The number of animals in each gender was small and, therefore, solid conclusions cannot be drawn. The results suggest that gender differences may be worth exploring concerning the effect of diet supplementation with protein hydrolysates on glucose intolerance.

**Figure 2:**
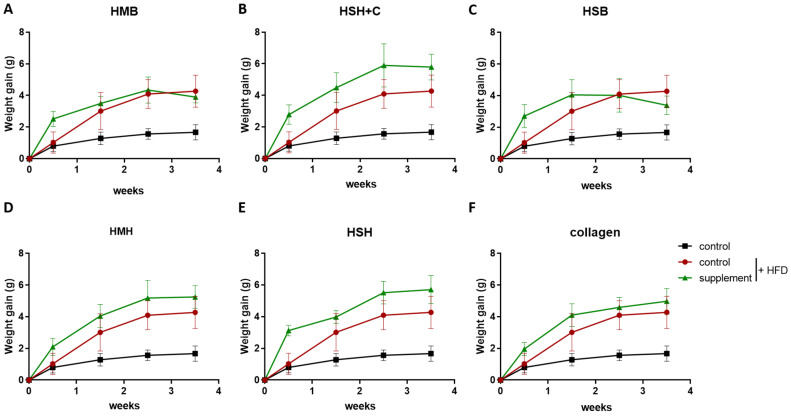
The effect of fish-derived hydrolysates in weight accumulation during a high-fat diet (**A**–**F**). Black color indicates the group of mice consuming the lean diet, and red color indicates the group of mice consuming the high-fat diet used as control. Graphs represent mean ± SEM. A 2-way ANOVA test was performed. No statistical significance was observed.

Animals rapidly gain weight when they follow a diet rich in fat. When mice were fed a high-fat diet for 3.5 weeks, they indeed put on considerably more weight than the lean diet group (Figure 2). The supplemented HFD groups show similar weight gain compared to the HFD control group or a non-statistically significant tendency towards elevated weight accumulation, especially at the very start of the feeding period (Figure 2). That could be explained by the slightly higher calorie number of the extracts compared to the soy supplement of the control group due to their high protein content. Nevertheless, glucose tolerance was not affected, as none of the somewhat fattier groups exhibited any effect in GTT. Animal feed consumption was nearly identical between the HFD groups and lower than the lean-diet group, probably because of the calorie-denser and more fulfilling diet of the former (Supplementary Figure S2). The final measurement was made on the day of sacrifice, for which the animals had to fast in order to undergo the GTT test, which explains the slight weight loss at the end of the experiment in some of the groups. Interestingly, the HSH group, which exhibited increased insulin sensitivity (Figure 1), accumulated almost as much fat or slightly more than the other HFD groups; therefore, an improvement in insulin sensitivity can be noted independently of an anti-obesity effect of the HSH extract.

**Figure 3:**
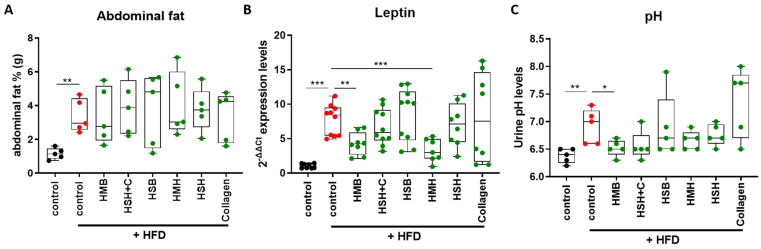
The effect of fish-derived hydrolysates in abdominal fat accumulation (**A**), Leptin gene expression (**B**), and urine pH (**C**) during a high-fat diet. A Mann–Whitney *t*-test was performed. * *p* < 0.05, ** *p* < 0.01, *** *p* < 0.001.

During excess weight gain, abdominal adipose tissue accumulates fat and may contribute to insulin resistance. Male epididymal and female perigonadal adipose tissues were extracted and weighed at the end of the experiment. All animals following the HFD had significantly increased visceral fat accumulation compared to the lean diet control group (Figure 3A). No statistically significant effect concerning abdominal fat accumulation was observed among the different nutritional supplement-consuming groups compared to the HFD control group.

Leptin is an important adipokine with a central homeostatic role, as it regulates, among other, satiety by acting in the brain, energy balance, and insulin sensitivity [23]. A common trait of diet-induced obesity is leptin resistance, characterized by increased circulating levels of leptin to compensate for the deteriorated leptin sensitivity [24]. In a state of obesity, the inhibitory leptin-induced signals on the appetite are diminished, consequently leading to hyperphagia, even in the presence of high circulating leptin levels [25]. As expected, when obese individuals were injected with additional leptin, obesity was not reversed [26]. Since leptin is primarily synthesized and secreted by the white adipose tissue, the excised male epididymal and female perigonadal fat tissues were homogenized to measure mRNA levels of leptin. The obese HFD groups exhibited considerably higher *leptin* gene expression compared to the lean diet control group, as expected. The expression of leptin is analogous to the energy stores [27], as enlarged adipocytes would express more leptin. Therefore, reduced leptin levels in the HMB and HMH groups that were observed independently of abdominal fat mass may be due to changes in adipocyte size and amelioration of glucose intolerance.

While the role of leptin in obesity is highly complicated and has not yet been completely elucidated, it is quite possible that leptin resistance is not only an important consequence of obesity, but also a serious risk factor for it [28]. Therefore, the significantly decreased levels of leptin expression of the HMB and the HMH groups could indicate higher leptin sensitivity and possibly leptin-mediated protection from obesity. Whereas the HMH group had a tendency for improved glucose tolerance, it is likely that a longer supplementation period was needed for a robust physiological effect to emerge.

Urine pH is affected by diet and related metabolites, and supplementation with HFD resulted in a significant increase in urine pH. This effect was reversed when the diet was supplemented with HMB (Figure 3C), suggesting metabolic changes following supplementation with the specific fish protein hydrolysate, which may also be due to changes in the gut microbiome, a major source of metabolites.

### 2.3. Collagen Supplementation Alters Microbiome Consistency

**Figure 4:**
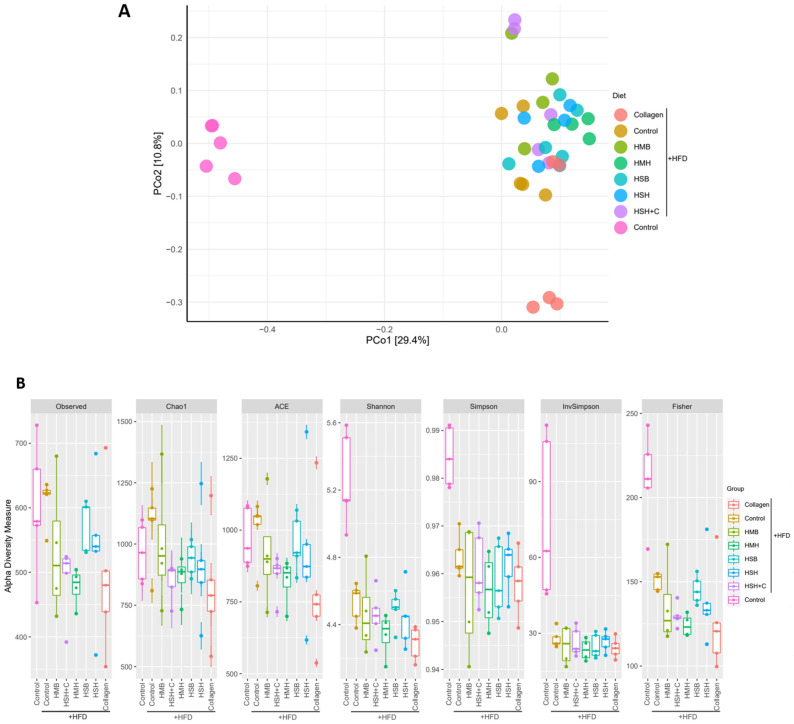
The effect of fish-derived protein hydrolysates on intestinal bacterial diversity. (**A**) PCoA and (**B**) Alpha Diversity analysis.

The gut microbiome, reaching up to 100 trillion cells, is composed of up to 150 times the number of genes of a human [29], rendering the latter capable of physiological functions that would normally not be able to be executed, as well as protected from or prone to a number of pathologies, depending on its composition. Multiple aspects of host metabolism and adiposity are modulated by the gut flora, for example, through the regulation of host gene expression and metabolic or inflammatory signaling pathways, as well as through affecting the behavior of the host via the gut–brain axis [30]. The gut microbiome is, in turn, shaped by a number of factors, including dietary components. The intestinal microbiota is altered in obesity and has been highlighted as a critical contributor to the development of obesity, diabetes, and metabolic syndrome [31]. Therefore, we examined the role of fish-derived protein hydrolysates in restoring the HFD-induced disturbances of the gut microbiome’s composition. Fecal samples were collected from animals following an HFD enriched with 5% fish sidestream-derived protein hydrolysates for 3.5 weeks, fecal DNA was subsequently extracted, and amplicon metabarcoding analysis was performed through 16S rRNA gene sequencing. Principal Coordinates Analysis (PCoA) was conducted based on the identified OTUs, to access the genetic clustering of the microbiome of the different diet groups (Figure 4A).

As anticipated, significant differences were observed between the lean diet and the HFD groups in PCoA. In particular, lean diet-fed animals and those following HFD formed two distinct clusters (Figure 4A). It is notable that the HFD groups were further clustered into three subgroups. The larger cluster contained the HFD control diet group, those supplemented with the HSB, HMH, and HSH extracts, the males fed with HSH + C and HMB supplements, and the females supplemented with collagen (center-right of the graph). PCoA clustered the female members of the HSH + C and HMB diet groups together (top-right of the plot), whereas males of the collagen diet group clustered together (bottom-right of the plot), indicating a gender-specific effect of the corresponding supplements. Although mice supplemented with the HSH extract exhibited an improvement in glucose tolerance (Figure 1E), PCoA displayed no significant changes in the gut microbiome compared to the HFD control group. Furthermore, the lean diet-fed control group was found to be the most diverse in terms of species richness based on the indexes Shannon, Simpson, InvSimpson, and Fisher, concerning unique OTUs, whereas the HFD groups exhibited a significantly lower alpha diversity measure (Figure 4B), as expected. No intense differences were noted in the Observed, Chao1, and ACE indexes. The Collagen group, whose male mice clustered separately in PCoA, had the least diverse bacterial profile when measuring unique OTUs, confirming the results obtained from a previous study in which we tested a different series of fish-derived supplements containing collagen [32].

**Figure 5:**
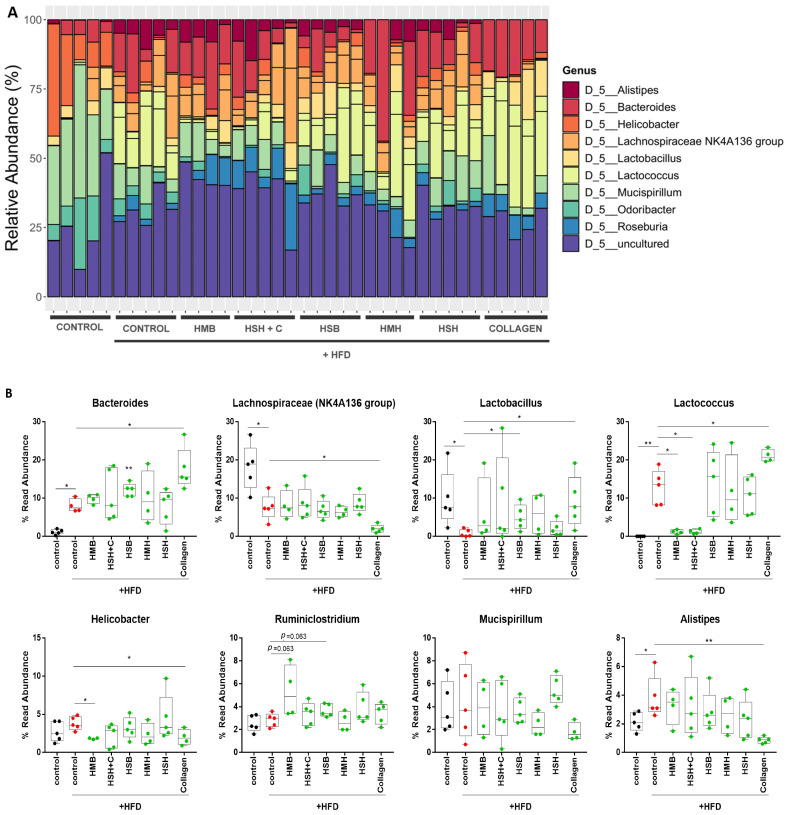
The effect of fish-derived dietary supplements on gut microbiome top genera composition. (**A**) Normalized bar plots of genera abundances (%) based on the identified OTUs; (**B**) Boxplots representing the abundance of different genera following diet supplementation. Mann–Whitney *t*-test was performed. * *p* < 0.05, ** *p* < 0.01.

Taxonomic classification of the identified OTUs revealed some important differences, mainly among the genera comprising the lean diet-fed mice and the HFD-fed mice (Figure 5). Collagen supplementation induced the colonization of *Bacteroides*, *Lactobacillus*, and *Lactococcus*. *Bacteroides* are beneficial bacteria that constitute a significant proportion of the mammalian gastrointestinal microbiome, are associated with diets with a considerable content of protein and animal fat, and have been suggested to halt the progression of obesity-related metabolic phenotypes [33], whereas the genera *Lactobacillus* and *Lactococcus* include well-studied probiotic bacteria found in a variety of mammals [34]. *Lachnospiraceae* occupy a substantial portion of the gastrointestinal microbiota of ruminants, as they metabolize a number of plant polysaccharides [35]. Therefore, they are expected to be more abundant in the lean-diet group than in those fed an HFD supplemented with protein hydrolysates of animal origin (Figure 5B). Moreover, it has been indicated that they may contribute to the emergence of diabetes in obese mice, and, interestingly, fish-derived collagen supplementation deduced their presence [36] (Figure 5B). Collagen also decreased the abundance of *Helicobacter*, which includes species that are pathogenic to humans, causing gastrointestinal diseases such as peptic ulcers, gastric cancer, Crohn’s disease, and ulcerative colitis [37], as well as the relative proportion of *Alistipes*, a newly identified bacterial genus with conflicting evidence regarding its role, as both beneficial and harmful effects have been documented [38]. Furthermore, the HSB group exhibited reduced *Lactobacillus* abundance, the HMB and HSH + C diet groups displayed decreased *Lactococcus* colonization but increased *Ruminiclostridium* abundance, while the HMB group alone showed lower *Helicobacter* presence. In Supplementary Figure S3, additional results of the intestinal microbiome composition of the top phyla, classes, orders, families, and species are presented. Overall, the most robust gut microbiome-modulatory effect was exhibited by collagen supplementation of fish origin, which elevated the abundance of beneficial bacteria and restricted the presence of harmful bacteria in mice with obesity and insulin resistance. Further research could unravel the potential role of fish-derived collagen supplementation in lean diet-fed mice.

## 3. Materials and Methods

### 3.1. Protein Hydrolysates

Enzymatic protein hydrolysates based on mackerel heads (HMH), mackerel backbones (HMB), salmon heads (HSH), and salmon backbones (HSB) were produced according to Aspevik et al., 2021 [17] in the pilot raw material processing plant of Nofima AQUAFEED Technology Center (Fyllingsdalen, Norway). Flounder skin collagen was kindly provided by Seagarden (Karmøy, Norway). The chemical properties of protein hydrolysates based on mackerel and salmon are shown in Daskalaki et al., 2021 [39]. The chemical properties of protein hydrolysate of collagen are shown in Table S1. All other chemicals were of analytical grade.

### 3.2. Animal Maintenance

C57BL/6 mice were maintained in a pathogen-free animal facility in the Medical School of the University of Crete, Heraklion, 12 h day/night cycle and 21–23 °C conditions. All procedures were conducted in compliance with protocols approved by the Animal Care Committee of the University of Crete, School of Medicine (Heraklion, Crete, Greece) and the Veterinary Department of the Region of Crete under license number 269904 (Heraklion, Crete, Greece).

### 3.3. Animal Protocols

Each diet group of mice consisted of 5 (12-week-old) animals (3 males and 2 females) and was introduced to a high-fat diet supplemented with fish-derived extracts for 3.5 weeks. To familiarize mice with the smell and taste of fish-derived supplements, they were introduced to the extracts for one week prior to the initiation of the experiment. The final concentration of every fish-derived supplement was 5% *w*/*w*. As a control supplement, 5% *w*/*w* of soy protein was used since soy protein is the main source of protein in rodent diets. Additionally, a group of mice consuming a lean diet supplemented with 5% *w*/*w* soy protein was used as a control. Mice’s weight and diet consumption were monitored twice per week. For the glucose tolerance test (GTT), mice were fasted overnight, and then glucose measurements were conducted to determine basal glucose levels, referred to as timepoint 0. Then, mice were injected intraperitoneally with sterile 35% dextrose (100 μL dextrose/35 g mice), and blood glucose levels were determined 30, 60, and 120 min post-injection. All glucose measurements were conducted using TRUEresult^®®^ Twist meter. For the urine pH measurement, each mouse, prior to euthanizing, was put in a reparation box for urine collection. pH values were determined using URS-10T Reagent Strips for Urinalysis reagent test strips.

### 3.4. cDNA Synthesis-Real Time PCR

Mice were euthanized through cervical dislocation, and immediately after animal sacrifice, epididymal white fat tissue (eWAT) was collected, weighed, and stored at −80 °C for further analyses. For RNA extraction, total eWAT was homogenized in Trizol^TM^ reagent using a mechanical homogenizer. RNA extraction was performed according to the manufacturer’s instructions. Then, 500 ng of total RNA was reverse transcribed using PrimeScript^TM^ RT Master Mix (Perfect Real Time) (RR036A, TaKaRa, Kusatsu, Japan) following the manufacturer’s instructions. Two-step quantitative PCR was performed in technical duplicates using the Kapa SyBr^®®^ Fast Universal kit (KK4618, Merck, Rahway, NJ, USA) on a 7500 Fast Real-Time PCR Instrument (Applied Biosystems^®®^, 4351106, Waltham, MA, USA) with 96-well Block Module as follows: start step 95 °C for 3 min and then 40 cycles of 95 °C for 10 s and 60 °C for 30 s. *18S* rRNA was used as an internal control gene. Primer sequences were designed as follows: 18S forward 5′-ATGGTCAACCCCACCGTGT-3′ and reverse 5′-TTCTGCTGTCTTTGGAACTTTGTC-3′, leptin forward 5′-TCACACACGCAGTCGGTATC-3′ and reverse 5′-GCACATTTTGGGAAGGCAGG-3′. Data analysis was conducted using mRNA levels expressed as relative quantification (RQ) values, which were calculated as RQ = 2^(−DDCt)^, where DCt is (Ct (gene of interest)—Ct (housekeeping gene)).

### 3.5. Gut Microbiome Analysis

Immediately after animal sacrifice, fecal samples were collected from the large intestine, caecum, and terminal ileum of the small intestine and stored at −80 °C for further analysis. Then, approximately 200 mg of stool was used to perform DNA extraction using a Nucleospin DNA stool kit (74047250, Macherey-Nagel, Düren, Germany) according to the manufacturer’s instructions. Bacterial community analysis was performed by amplifying and sequencing the V3–V4 hypervariable regions of the 16S rRNA gene (≈460 bp). Libraries were constructed according to Illumina’s protocol for 16S metagenomic analyses described in ‘Illumina’s 16S Metagenomic Sequencing Library Preparation’ (15044223 B) manual. For the amplification of the V3–V4 regions, primers were selected from Klindworth et al., 2013 [40] and further modified by adding an Illumina adapter sequence at the 5′ end of each primer. The size and quality of the constructed libraries were evaluated using the Fragment Analyzer 5200 system (Agilent Technologies Inc., Santa Clara, CA, USA) and the DNF-915-33 kit. Sequencing was conducted on a MiSeq platform (Illumina Inc., San Diego, CA, USA) using the MiSeq^®®^ reagent kit v3. For the assessment of the prokaryotic load, bioinformatic analysis was performed using the QIIME2 pipeline (Quantitative Insights Into Microbial Ecology, https://qiime2.org/, accessed on 10 May 2023) (v.2018.2) [41]. Raw reads were imported into QIIME2, and adapter sequences were removed using the cutadapt plug-in [42]. Adapter-trimmed paired-end sequences were joined with the vsearch plug-in [43] and quality-filtered with a minimum quality score of 30. Subsequently, the vsearch plug-in was employed to dereplicate the filtered sequences, cluster them into OTUs (Operational Taxonomic Units) at 99% nucleotide homology, using the open-reference method and the SILVA 132 database [44], and remove chimeric sequences from the data. Taxonomy was assigned to the OTUs by aligning them against the SILVA 132 database at 99% sequence identity using the BLAST plug-in [45]. Sequences classified as archaeal, chloroplastic, or mitochondrial, along with sequences that were not assigned a taxonomy, were then removed from the data. The final OTU table was imported into R version 3.6.0 [46], and results visualization was performed using the phyloseq [47], ampvis2 [48], and ggplot2 [49] R packages. Barplots depicting OTU counts were normalized to 100% as abundance estimations within each sample.

### 3.6. Statistical Analysis

Data are presented as mean ± SEM. Graphs were designed, and statistical analysis was performed using Graphpad Prism 7.0. A Mann–Whitney *t*-test was performed to test the statistical analysis of each supplement to the control diet as well as 2-way ANOVA. Differences with a *p*-value < 0.05 are considered significant (* indicates *p* < 0.05, ** indicates *p* < 0.01, *** indicates *p* < 0.001).

## 4. Conclusions

This work demonstrates the pivotal role of fish side stream-derived protein hydrolysates in alleviating glucose tolerance in high-fat diet-induced obesity in mice, whereas collagen supplementation induced intestinal microbial changes favoring the colonization of beneficial bacteria. In particular, diet supplementation with HSH supplement ameliorated insulin resistance in high-fat-induced obese mice, while diet supplementation with HMB and HMH nutritional supplements eliminated leptin expression in the adipose tissue independently of abdominal fat accumulation in obese mice. Intestinal microbiome beneficial alterations were most prominent in collagen supplemented group. The health-promoting properties of bioactive peptides provide new evidence for an important, underexplored source of dietary products with health-promoting properties.

## Figures and Tables

**Table 1:** List of tested supplements and their composition.

Supplement	Composition
HMB	Hydrolysate Mackerel Backbone
HSB	Hydrolysate Salmon Backbone
HSH	Hydrolysate Salmon Heads
HSH + C HMH	50% Hydrolysate Salmon Heads + 50% Collagen Hydrolysate Mackerel Heads
Collagen	100% Collagen

## Data Availability

All raw data are readily available upon request.

## Supplementary Materials

The following supporting information can be downloaded at: https://www.mdpi.com/article/10.3390/md21060343/s1, Figure S1: Monitoring the effect of HMB diet supplementation in male mice in high-fat diet-induced insulin resistance; Figure S2: Measuring Feed consumption supplemented with the indicated protein hydrolysate in a 2-week period (A–F); Figure S3: Phylogenetic analysis of different taxa in supplement consuming groups.
