# Supplementary material for: Fish-Derived Protein Hydrolysates Increase Insulin Sensitivity and Alter Intestinal Microbiome in High-Fat-Induced Obese Mice
Source: Mar Drugs. 2023 Jun 2;21(6):343. doi: 10.3390/md21060343 (PMC10302332; doi:10.3390/md21060343)
Supplement: Supplementary file 1 [file marinedrugs-21-00343-s001.zip › marinedrugs-2418219-supplementary.pdf]

marine drugs

Supplementary Material for

Fish-derived protein hydrolysates increase insulin sensitivity and alter intestinal microbiome in high-fat induced obese mice

Maria G. Daskalaki [1,2,†], Konstantinos Axarlis [1,2,†], Antiopi Tsoureki [3], Sofia Michailidou [3], Christina Efraimoglou [1,2], Ioanna Lapi [1,2], Ourania Kolliniati [1,2] , Eirini Dermitzaki [1,2], Maria Venihaki[1], Katerina Kousoulaki[4], Anagnostis Argiriou[3,5] and Christos Tsatsanis [1,2,*]

[1] Laboratory of Clinical Chemistry, Medical School, University of Crete, Heraklion 70013, Greece; konax@outlook.com (KA); m.daskalaki@med.uoc.gr (MGD); christinaefr96@gmail.com (CE); raliakolliniatis21@gmail.com (OK); iwanna_lapi@hotmail.com (IL); tsatsani@uoc.gr (C.T.)
[2] Institute of Molecular Biology and Biotechnology, FORTH, 71100 Heraklion, Greece
[3] Institute of Applied Biosciences (INAB), CERTH, Thermi-Thessaloniki GR-57001, Greece; adatsoureki@certh.gr (AT), sofia_micha28@certh.gr (SM), argiriou@certh.gr (AA)
[4] Department of Nutrition and Feed Technology, Nofima AS, 5141 Bergen, Norway; katerina.Kousoulaki@Nofima.no (KK)
[5] Department of Food Science and Nutrition, University of the Aegean, Myrina, 81400, Lemnos, Greece;
† These authors contributed equally to this work.
* Correspondence: tsatsani@uoc.gr, Tel.: +30-2810394833 (CT)

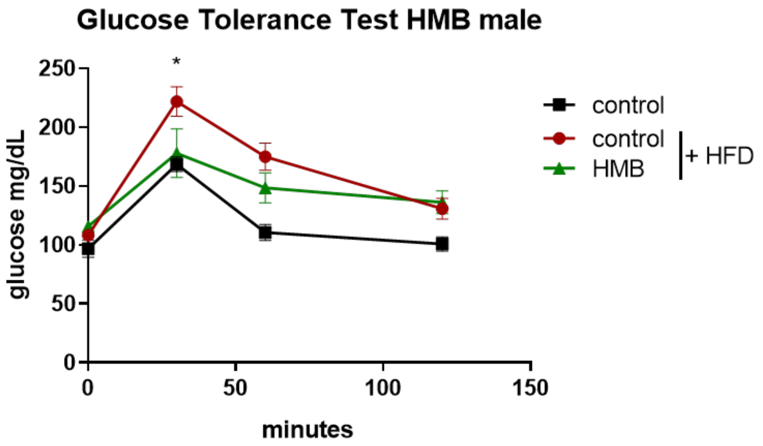

Figure S1. Monitoring the effect of HMB diet supplementation in male mice in high-fat diet induced insulin resistance. A-F. Black colour indicates the goup of mice consuming the lean diet, red colour indicates the group of mice consuming the high-fat diet used as control. Graph represent mean ± SEM. 2-way ANOVA test was performed.* p < 0.05, ** p < 0.01, *** p < 0.001

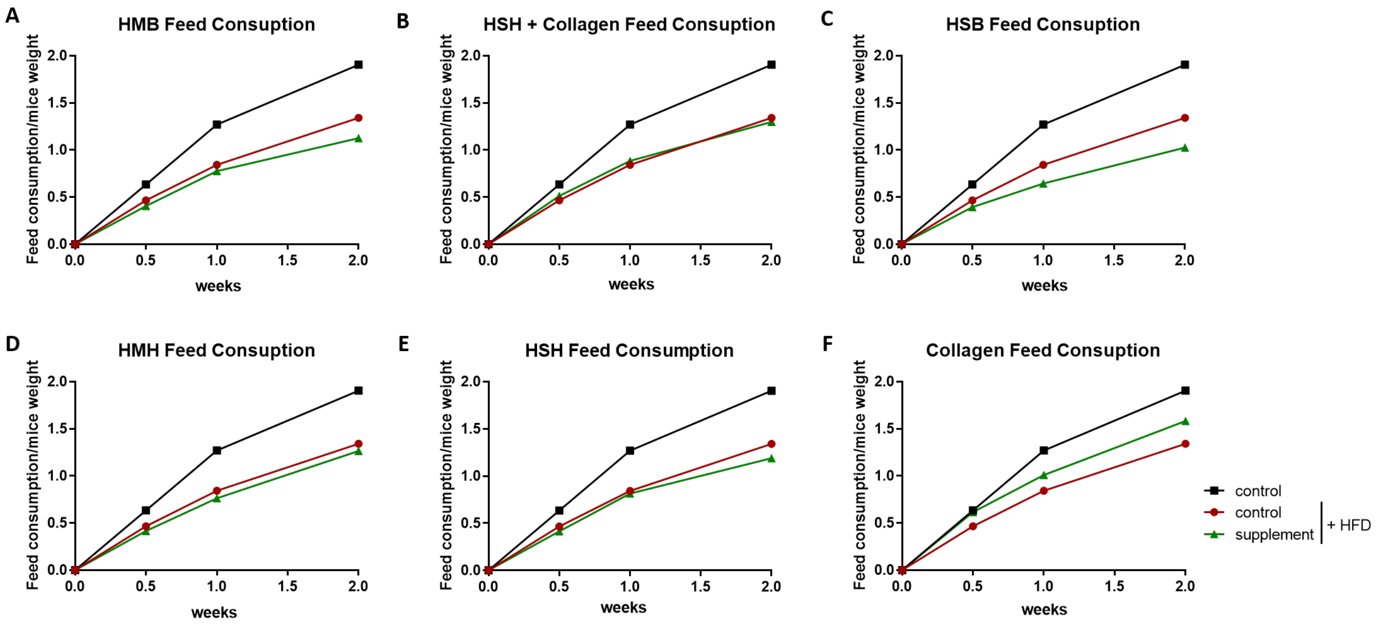

Figure S2. Measuring Feed consumption supplemented with the indicated protein hydrolysate in a 2-week period (A-F). Black colour indicates the group of mice consuming the lean diet, red colour indicates the group of mice consuming the high-fat diet used as control.

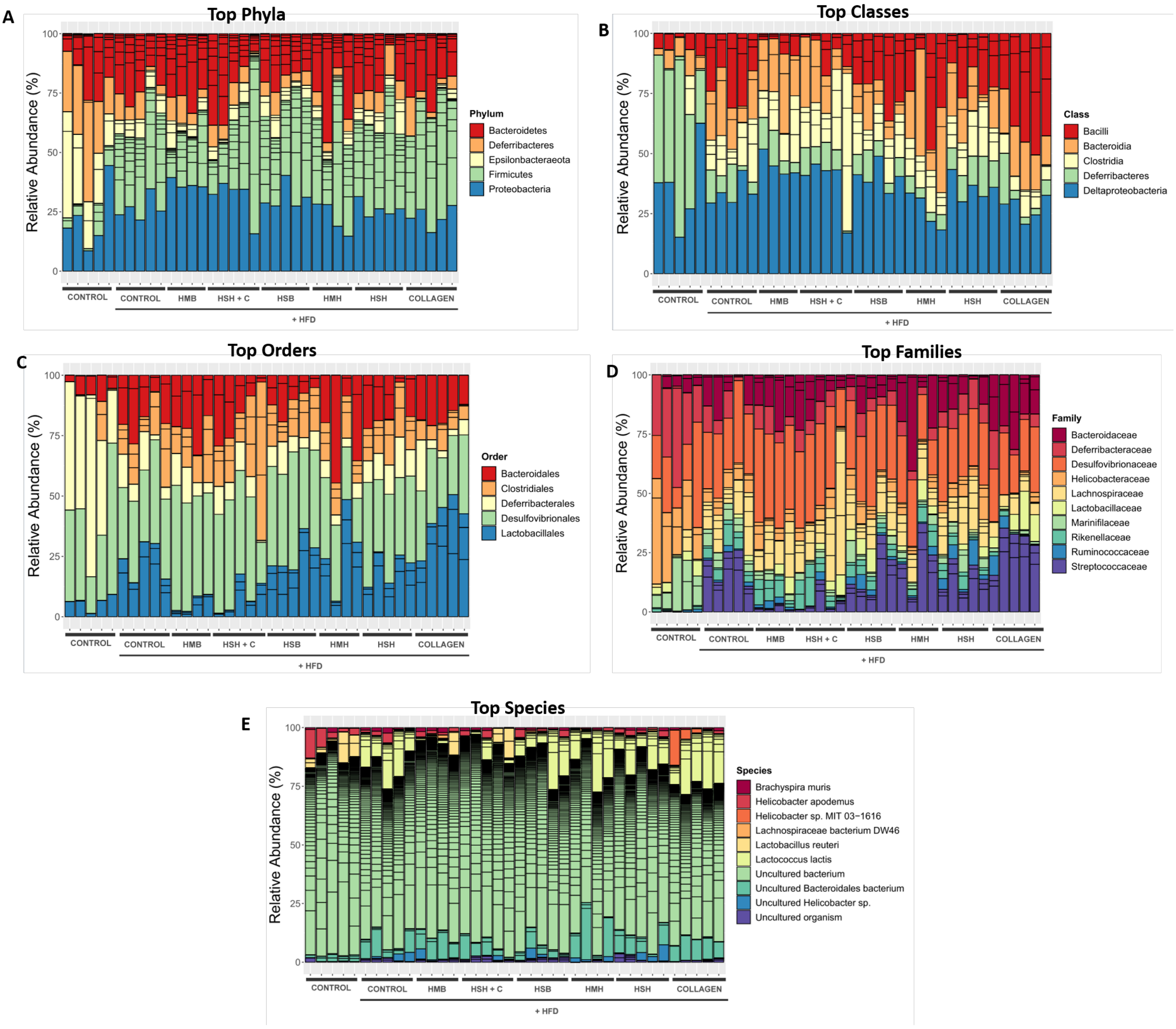

Figure S3. The effect of fish-derived protein hydrolysateson the intestinal microbiome composition of top A. phyla, B. classes, C. orders, D. families and E. species. Top genera are depicted in the main paper.
